# 2′-O-Methylperlatolic Acid Enhances Insulin-Regulated Blood Glucose-Lowering Effect through Insulin Receptor Signaling Pathway

**DOI:** 10.1155/2022/2042273

**Published:** 2022-04-23

**Authors:** Wang Yinghao, Guan Qiaoli, Liu Guanfu, Wu Xiaoyun, Wang Xuanjun, Sheng Jun

**Affiliations:** ^1^Key Laboratory of Puer Tea Science, Ministry of Education, Yunnan Agricultural University, Kunming, China; ^2^Scientific Observing and Experimental Station of Tea Resources and Processing in Yunnan, Ministry of Agriculture, Kunming, China; ^3^Department of Science, Yunnan Agricultural University, Kunming, China

## Abstract

**Purpose:**

Insulin receptor (InsR) sensitizers represent a new type of therapeutic agent for the treatment of diabetes, with 2′-O-methylperlatolic acid (2-O-M) being a potential InsR targeting drug. The purpose of this study was to determine whether 2-O-M functions as an activator of the insulin signaling pathway, regulating glucose hemostasis through the InsR and exerting a glucose-lowering effect in an animal model of diabetes.

**Methods:**

SPR-based analyses were used to detect the binding of different concentrations of 2-O-M to the InsR. The protein levels of IR-*β*, p-IR, AKT, and p-AKT in Hepa and C2C12 cell lines and liver and muscle tissues were determined by western blotting. Glucose uptake capacity was determined in C2C12 cells. Streptozotocin-induced diabetic mice were randomly divided into four groups: the control, insulin treated, 2-O-M treated, and combined insulin and 2-O-M treated. Mice were injected with 2-O-M or normal saline and the average blood glucose concentration after 120 min, and the serum levels of insulin, glucagon, and C-peptide were measured. Next, qRT-PCR was performed to detect the mRNA expression of genes involved in lipid and glucose metabolism in the liver and muscle tissues.

**Results:**

2-O-M binds to the extracellular domain of the InsR. Moreover, combination treatment with 2-O-M and insulin resulted in significant activation of the insulin signaling pathway *in vitro* and significant stimulation of the glucose uptake capacity of C2C12 myotubes. In mice with streptozotocin-induced diabetes, 2-O-M significantly prolonged the blood glucose-lowering effect of insulin, significantly reduced the secretion of exogenous insulin, and reduced the blood glucose concentration *in vivo*. In addition, treatment with 2-O-M alone significantly enhanced the phosphorylation of AKT in muscle tissue, which enhanced glucose uptake in C2C12 myotubes. Further, 2-O-M significantly increased glucagon secretion and enhanced liver gluconeogenesis to prevent hypoglycemia.

**Conclusion:**

2-O-M enhances the hypoglycemic effect of insulin through the insulin signaling pathway and can be used as a complement to insulin. This synergetic effect may lower the required dose of insulin and protect *β* cells.

## 1. Introduction

The incidence of diabetes has increased at an alarming rate, making it a major global public health concern. A review of population health in 110 countries from 1980 to 2014 identified 366 million patients with diabetes in 2011, and this number is expected to increase to 552 million by 2030 [1]. There are four types of diabetes: type 1 diabetes (insulin deficiency), type 2 diabetes (insulin resistance), specific-type diabetes, and gestational diabetes [2]. High circulating concentrations of glucose caused by diabetes can lead to various chronic diseases, including retinopathy, diabetic nephropathy, and chronic cardiovascular disease [3–5]. Diabetes also increases the risk of cancer [6]. In addition, diabetes imposes a heavy economic burden on individuals and households as well as on healthcare systems [7].

Effective pharmacological glycemic control is the key to treating diabetes [8]. Currently, the main drugs used to treat diabetes include insulin, insulin analogues, metformin, sodium-glucose cotransporter-2 (SGLT2) inhibitors, and natural compounds [9–11]. Medicinal use of natural compounds in the treatment and prevention of diseases, including diabetes, has a long history compared to conventional medicines. Moreover, herbal medications may be used as effective and sustainable alternatives to treat diabetes [10]. Many plants have proven antidiabetic activity, with their main ingredients being polyphenols [12]. Polyphenol compounds found in many plants can enhance insulin sensitivity and reduce blood glucose in animal models of diabetes [13–17]. Therefore, research into new antidiabetic drugs from natural compounds is becoming increasingly relevant in the search for novel treatments for diabetes.

The InsR is one of the most important targets for diabetic drug discovery [18, 19]. InsR sensitizers can bind to the InsR to activate the insulin pathway independent of insulin; thus, InsR sensitizers have the potential to alleviate insulin resistance and minimize the risk of hypoglycemia [19]. InsR sensitizers increase insulin sensitivity for patients with type 2 diabetes and lower glucose levels for patients with type 1 disease. Given these attributes, InsR sensitizers represent an opportunity to develop a new type of therapeutic drug to treat diabetes. However, only two InsR sensitizers, TLK19781 [19] and TLK16998 [20, 21], have been identified. Because insulin sensitizers offer so many treatment advantages, finding new examples of these drugs in natural compounds may play a major role in improving the quality of life of patients with diabetes.

In this study, surface plasmon resonance (SPR) analysis was used to determine the binding affinities between natural compounds and the extracellular domain of the InsR. The polyphenolic compound, 2′-O-methylperlatolic acid (2-O-M), is a monoamine oxidase B inhibitor extracted from *Pertusaria parasommerfeltii*, and it directly binds with the InsR. The combination of 2-O-M with insulin enhances the function of insulin-induced glucose-lowering effect through activation of insulin signaling pathway in both type 1 diabetes mice and type 2 diabetes mice models. In this study, the streptozotocin- (STZ-) induced diabetic mice represent a model of type 1 diabetes, while *db/db* mice represent a model of insulin resistance induced type 2 diabetes. We found that 2-O-M regulates glucose homeostasis by stimulating the insulin signaling pathway in liver and muscle tissues, which synthesize glycogen, lipids, and gluconeogenesis genes.

## 2. Materials and Methods

### 2.1. Animals

All animal care and animal experiments in this study were approved by the animal ethics committee of Yunnan Agricultural University (no. 202010057). Mature and healthy BALB/c mice and *db/db* mice aged 6–8 weeks were purchased from Cawens Lab Animal Co. (Changzhou, China). The mice were individually housed in an environmentally controlled room (ventilated, 22°C, relative humidity of 55% ± 5%) with a 12-hour light-dark cycle. Mice were given free access to water and food.

Two weeks before commencing the modeling experiment, the BALB/c mice were fed a high-fat diet (D12492, research diet) with fat making up 60% of the total caloric intake. These mice were fasted overnight and then injected with 55 mg/kg of streptozotocin (Sigma-Aldrich, St. Louis, USA) for 3 consecutive days. After 7 days, blood glucose was measured. If the blood glucose was ≥15 mmol/L, the type 2 diabetes model was considered successfully established [22]. Diabetic mice were randomly divided into four groups (4-8 mice in each group): the control group, the insulin group, the 2-O-M group, and the combined (insulin+2-O-M) treatment group. Insulin was injected subcutaneously at a dose of 0.5 U/kg, and 2-O-M was injected into the tail vein at a concentration of 1 mg/kg. We purchased 2-O-M (95.0% purity) from BioBioPha (Kunming, China), dissolved in dimethyl sulfoxide (DMSO) to make a 10 mmol/L stock solution, and stored at −20°C in the dark until the tail vein injections were administered.

Diabetic *db/db* mice were randomly divided into four groups (6 mice in each group): the control group, the insulin group, the 2-O-M group, and the combined (insulin+2-O-M) treatment group. The *db/db* mice fasted overnight before the experiment. Insulin was injected subcutaneously at a dose of 1 U/kg, and 2-O-M was injected into the tail vein at a concentration of 1 mg/kg.

### 2.2. Cell Culture and Treatments

A mouse hepatocyte cell line, Hepa 1-6, and a skeletal muscle cell line, C2C12, were maintained in Dulbecco's modified Eagle's medium (DMEM) supplemented with 10% fetal bovine serum (FBS; Gibco, Waltham, USA) and 1% penicillin-streptomycin liquid (Solarbio Life Science, Beijing, China). For differentiation, C2C12 cells were placed in 2% horse serum in DMEM for 6–7 days and incubated at 37°C under humidified conditions of 95% air and 5% CO_2_. Before each experiment began, the cells were washed with phosphate-buffered saline (PBS) buffer and were maintained in DMEM without FBS for at least 4 h. For measurement of protein phosphorylation, the cells were treated with 1 nM insulin or 4 *μ*M 2-O-M for 20 min before cell lysates were collected for western blotting. Insulin was used as the positive control.

### 2.3. Cell Viability Assay

The effects of 2-O-M on cell growth were evaluated using the 3-(4,5-dimethylthiazol-2-yl)-2,5-diphenyltetrazolium assay. Briefly, Hepa 1-6 cells were seeded at 1.5 × 10^4^ cells/well in a 96-well plate, cultured overnight, and then treated with different concentrations of 2-O-M (1–16 *μ*M) or 1 nM insulin for 24 h. After the supernatant was removed, DMSO was added to the wells, and the optical density at 492 nm was measured with a microplate reader (FlexStation 3; Molecular Devices, Sunnyvale, USA). Cell viability was normalized to that of control cells.

### 2.4. Real-Time Quantitative Polymerase Chain Reaction Analysis

TRIzol (TransGen Biotech, Beijing, China) was used to extract total RNA from muscle and liver tissues. Next, cDNA was synthesized in accordance with the manufacturer's instructions using the PrimeScript RT reagent kit with gDNA Eraser (Takara, Japan). PCR detection and quantification were performed using TB Green® Premix Ex Taq™ II (Tli RNaseH Plus) (Takara, Japan) and the Roche 480 (Roche, Basel, Switzerland) fast real-time PCR system. Gene expression levels were calculated using the 2^−*ΔΔ*CT^ method and normalized to *β*-actin expression in liver tissue and normalized to *α*-tubulin expression in muscle tissue. All primer sequences are listed in Table 1.

### 2.5. Western Blotting Analysis

RIPA buffer and supporting PMSF (Solarbio Life Science, Beijing, China) were used to extract total protein content from Hepa cells, C2C12 cells, and liver and muscle tissues. Protein concentration was measured using the BCA protein determination kit (Beyotime Biotechnology, Shanghai, China). An equal mass (60 *μ*g) of each protein sample was loaded into each well of an 8% sodium dodecyl sulfate-polyacrylamide gel electrophoresis (SDS-PAGE) gel. Proteins were then transferred to a 0.45 *μ*m PVDF membrane, and nonspecific binding to the membrane was blocked by incubation in 5% (*w*/*v*) skimmed milk powder in1X TBST for 1 h. Membranes were then incubated with the indicated primary antibodies at 4°C overnight with shaking: anti-insulin receptor *β*, anti-phospho-insulin receptor *β*, anti-Akt, anti-phospho-Akt, and anti-*β*-tubulin. Antibodies were recovered, and membranes were washed three times for 5 minutes each with Tris buffer containing Tween 20 (TBST). The washed immunoblots were incubated with horseradish peroxidase-conjugated secondary antibody diluted in 5% skimmed milk powder in 1X TBST at room temperature with shaking for 1 h. Immunoblots were then visualized using the UltraSignal Ultra-Sensitive ECL Chemiluminescence Substrate (4A Biotech, Beijing, China), and *β*-tubulin staining was used as the loading control.

### 2.6. SPR Studies

SPR experiments were performed using a Biacore S200 instrument (Biacore, GE Healthcare) at 25°C. The extracellular domain of the InsR (10 *μ*g/mL in 10 mM sodium acetate, pH 4.5) was immobilized on an S CM5 sensor chip (GE Healthcare) using an amine coupling kit. The analytes (2-O-M at concentrations of 6.25–100 *μ*M, twofold dilution) were passed over the immobilized InsR on the sensor surface. The flow rate was set to 30 *μ*L/min with a binding time of 90 s and a dissociation time of 90 s. Kinetic and affinity analyses were performed using the Biacore S200 evaluation software (version 1.1, GE Healthcare).

### 2.7. Glucose Uptake Assay

C2C12 cells were treated with 4 *μ*M 2-O-M and with 1 nM insulin, and the glucose uptake capacity of the cells was measured with a glucose uptake kit (Promega, USA). The glucose uptake assay is based on the 2-deoxyglucose-6-phosphate (2DG6P) assay for the detection of glucose uptake by mammalian cells. When 2-deoxyglucose is added to the cells, it is transported into cells *via* the membrane GLUT2/GLUT4 protein and is rapidly phosphorylated in the same way as glucose. However, enzymes that further modify glucose-6-phosphate cannot modify 2-deoxyglucose-6-phosphate, and the reactants cannot penetrate the membrane, so the membrane-impermeable analyte accumulates in the cell. The culture media were removed from the cells, and the cells were lysed using acid culture. The detection of 2-deoxyglucose-6-phosphate produced after lysis positively reflected the glucose uptake capacity of the cells.

### 2.8. Detection of Serum Hormones

Diabetic mice were randomly divided into four groups as specified previously. After appropriate treatment, the blood glucose levels were tested at 120 min. Subsequently, the mice were euthanized, and the blood was collected from the canthus into microcentrifuge tubes with heparin sodium or an anticoagulant tube containing EDTA-2Na and aprotinin. The blood samples were centrifuged at 3,000 g for 20 min at 4°C, and the serum was kept frozen at −80°C until analysis. The levels of insulin, glucagon, and C-peptide were measured and analyzed using the relevant kits (Crystal Chem, USA) in accordance with the manufacturer's protocols.

### 2.9. Statistical Analysis

Statistical analysis of the experimental data was performed using SPSS 17.0 and GraphPad Prism 6. Data are presented as the mean ± SEM. Differences between groups were analyzed using one-way ANOVA; *P* < 0.05 was considered significant.

## 3. Results

### 3.1. 2-O-M Binds to InsR

**Figure 1:**
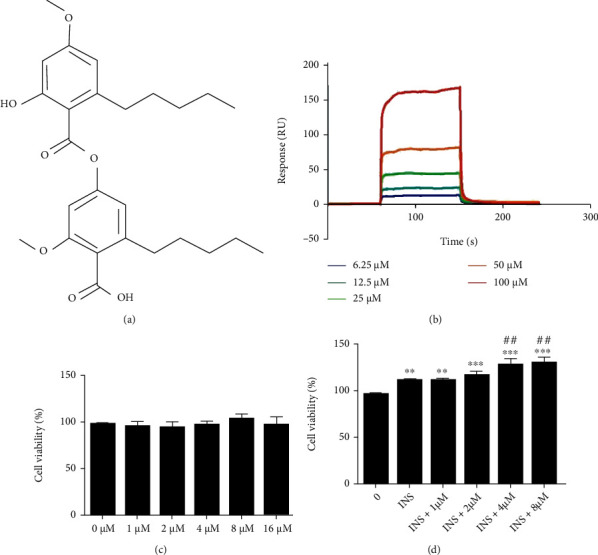
2′-O-Methylperlatolic acid (2-O-M) binds to the InsR. (a) Chemical structural of 2-O-M. (b) Binding affinity of 2-O-M to InsR. (c) Cytotoxic effects of 2-O-M on cells. The change in cell viability caused by 2-O-M in the presence of insulin. Data are shown as the mean ± SEM (*n* = 6/7 in each group). ^∗^*p* < 0.05; ^∗∗^*p* < 0.01; ^∗∗∗^*p* < 0.001 versus control. ^#^*p* < 0.05; ^##^*p* < 0.01; ^###^*p* < 0.001 versus insulin.

To determine whether 2-O-M (Figure 1(a)) interacts with the InsR, SPR-based analysis was used to detect the binding of 2-O-M to the receptor at different concentrations. The chemical structure of 2-O-M is shown in Figure 1(a). Different concentrations of 2-O-M (6.25–100 *μ*M) flowed over the InsR protein, which was immobilized on the CM5 chip. The result showed that 2-O-M may bind to the InsR, with a *K*_*D*_ of 88.72 *μ*M (Figure 1(b)).

To confirm the working concentration of 2-O-M on cells, we determined the cytotoxicity of 2-O-M on Hepa 1-6 cells, and the experiment revealed that the cell viabilities were not affected by 2-O-M (Figure 1(c)). To determine whether 2-O-M affected cell proliferation in the presence of 1 nM insulin, which mimics the concentration in circulation, Hepa 1-6 cells were incubated in a medium containing different concentrations of 2-O-M (0–8 *μ*M) and 1 nM insulin. In the presence of 1 nM insulin, 4 *μ*M and 8 *μ*M 2-O-M significantly enhanced cell viability; however, lower concentrations of 2-O-M (1 *μ*M and 2 *μ*M) did not improve cell viability (Figure 1(d)).

### 3.2. 2-O-M Enhanced Insulin-Activated Insulin Signaling Pathway and Stimulated Cellular Glucose Uptake

**Figure 2:**
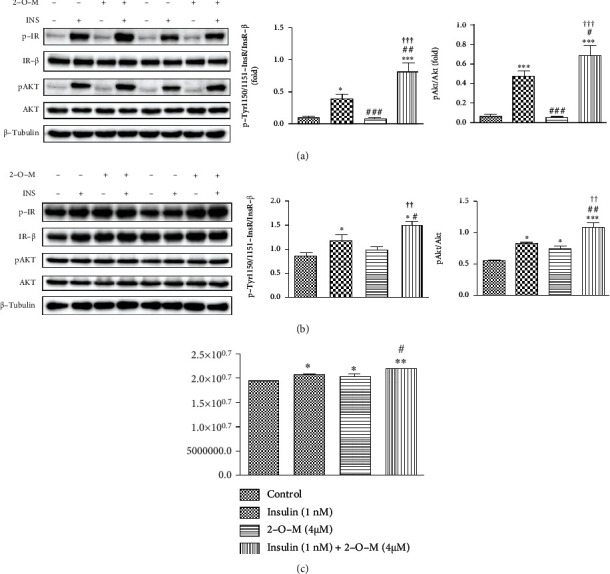
The combination of 2′-O-methylperlatolic acid (2-O-M) and insulin activates the insulin signaling pathway and stimulates cellular sugar uptake. (a) Expression of key proteins of the insulin signaling pathway in different groups of 2-O-M and insulin in Hepa 1-6 cells. (b) The expression of key proteins in the insulin signaling pathway in different groups of 2-O-M and insulin in C2C12 cells. (c) The effect of 2-O-M combined with insulin on glucose uptake capacity in C2C12 cells. Data are shown as the mean ± SEM (*n* = 6/7 in each group). ^∗^*p* < 0.05; ^∗∗^*p* < 0.01; ^∗∗∗^*p* < 0.001 versus control. ^#^*p* < 0.05; ^##^*p* < 0.01; ^###^*p* < 0.001 versus insulin. ^†^*p* < 0.05; ^††^*p* < 0.01, 2-O-M versus insulin+2-O-M.

Adverse effects from high doses of insulin include weight gain and excessive hypoglycemia. Low-dose insulin, combined with other medications, has become the primary treatment for diabetes [23]. To examine insulin receptor phosphorylation, Hepa 1-6 cells and C2C12-differentiated myocytes were both treated with insulin (1 nM) and 2-O-M (4 *μ*M). The autophosphorylation of InsRs and the phosphorylation of AKT significantly increased in the insulin (1 nM) group compared to the control group. Autophosphorylation of the receptor and the phosphorylation of AKT were significantly higher in the combined (insulin+2-O-M) treatment group compared to the insulin group (Figures 2(a) and 2(b)).

The goal of insulin signaling pathway activation is the promotion of the glucose uptake capacity of the cells. We determined the ability of the combination of insulin and 2-O-M to enhance glucose uptake. This combination significantly enhanced the glucose uptake capacity in C2C12 cells compared to the insulin-only treatment to cells (Figure 2(c)), and this result was consistent with the western blotting results shown in Figure 2(b).

### 3.3. 2-O-M Enhanced Insulin-Activated Hypoglycemic Effects in Diabetic Mice

The 6–8-week-old BALB/c mice were fed a high-fat diet (containing 60% fat kcal) for 2 weeks before attempting to establish the streptozotocin-induced model of diabetes. Mice were injected with 55 mg/kg of STZ for 3 consecutive days. Blood glucose levels ≥ 15 mmol/L were indicative of a successfully established disease model [22]. Changes in body weight and fasting blood glucose concentration are shown in Figure S1. Body weight decreased significantly in mice following STZ injection (Figure S1A), and blood glucose levels increased significantly 7 days after STZ injection (Figure S1B).

**Figure 3:**
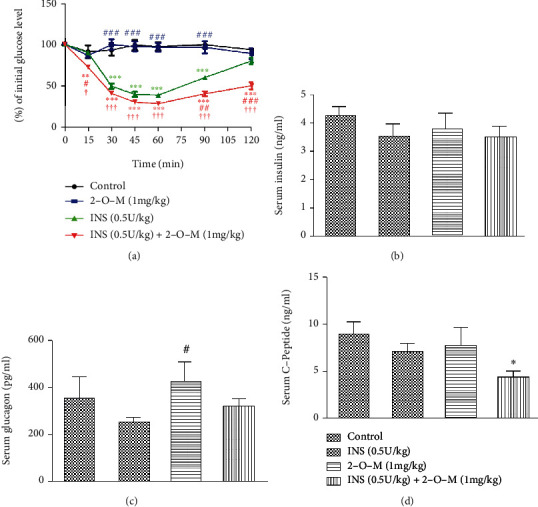
2′-O-Methylperlatolic acid (2-O-M) enhances the insulin-activated hypoglycemic effect in mice with STZ-induced diabetes. (a) Blood glucose change within 120 minutes. (b) Serum insulin level at 120 minutes. (c) Serum glucagon level at 120 minutes. (d) Serum C-peptide level at 120 minutes. Data are shown as the mean ± SEM (*n* = 4–8 in each group). ^∗^*p* < 0.05; ^∗∗^*p* < 0.01; ^∗∗∗^*p* < 0.001 versus control. ^#^*p* < 0.05; ^##^*p* < 0.01; ^###^*p* < 0.001 versus insulin.

**Figure 4:**
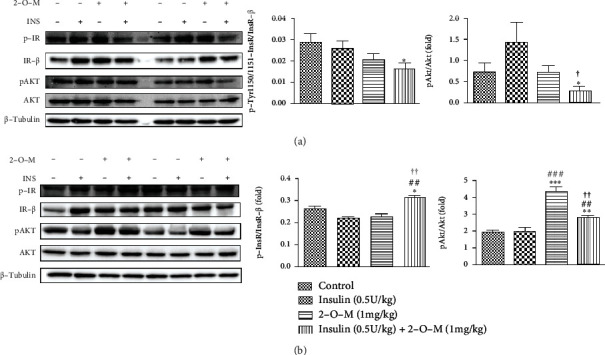
2′-O-Methylperlatolic acid (2-O-M) enhanced the insulin-activated hypoglycemic effect in *db/db* mice. (a) Blood glucose change within 120 minutes in *db/db* mice. (b) Serum insulin level at 120 minutes. Serum C-peptide level at 120 minutes. Serum glucagon level at 120 minutes. Expression of key proteins of the insulin signaling pathway in muscle. Data are shown as the mean ± SEM (*n* = 6 in each group). ^∗^*p* < 0.05; ^∗∗^*p* < 0.01; ^∗∗∗^*p* < 0.001 versus control. ^#^*p* < 0.05 versus insulin. ^†^*p* < 0.05, 2-O-M versus insulin+2-O-M.

To explore the hypoglycemic effect of 2-O-M *in vivo*, 2-O-M and insulin were injected into STZ-induced diabetic mice, and blood samples and tissues were collected for blood glucose analysis for up to 120 min after injection. Blood glucose levels revealed that the average blood glucose concentration was significantly lower at 60–120 min in the group that received both insulin and 2-O-M compared to the insulin and the control groups. Moreover, blood glucose in the insulin group had returned to its initial level (Figure 3(a)). Additionally, we injected 2-O-M and insulin into *db/db* mice and determined the blood glucose levels for 120 min. Our data revealed that the average blood glucose concentration was significantly lower at 90 and 120 min in the group that received both insulin and 2-O-M compared to the insulin-only group (Figure 4(a)).

We detected the serum levels of insulin, glucagon, and C-peptide in each group of STZ-induced diabetic mice and found no difference in serum insulin levels among the groups (Figure 3(b)). The average glucagon level in mice in the 2-O-M group was significantly higher than that in the insulin group (Figure 3(c)). The serum level of C-peptide in the combined treatment group was significantly lower than the level in the control group (Figure 3(d)), and there were no significant differences among the control, insulin, and 2-O-M groups (Figure 3(e)). In the same way, the levels of insulin, glucagon, and C-peptide in the serum of *db/db* mice were detected. There were no differences in the levels of serum insulin in each group (Figure 4(b)). The level of serum glucagon in the 2-O-M group was significantly higher than that in the 2-O-M group. The C-peptide level of the 2-O-M combined with the insulin group was also significantly lower than that of the control group.

**Figure 5:**
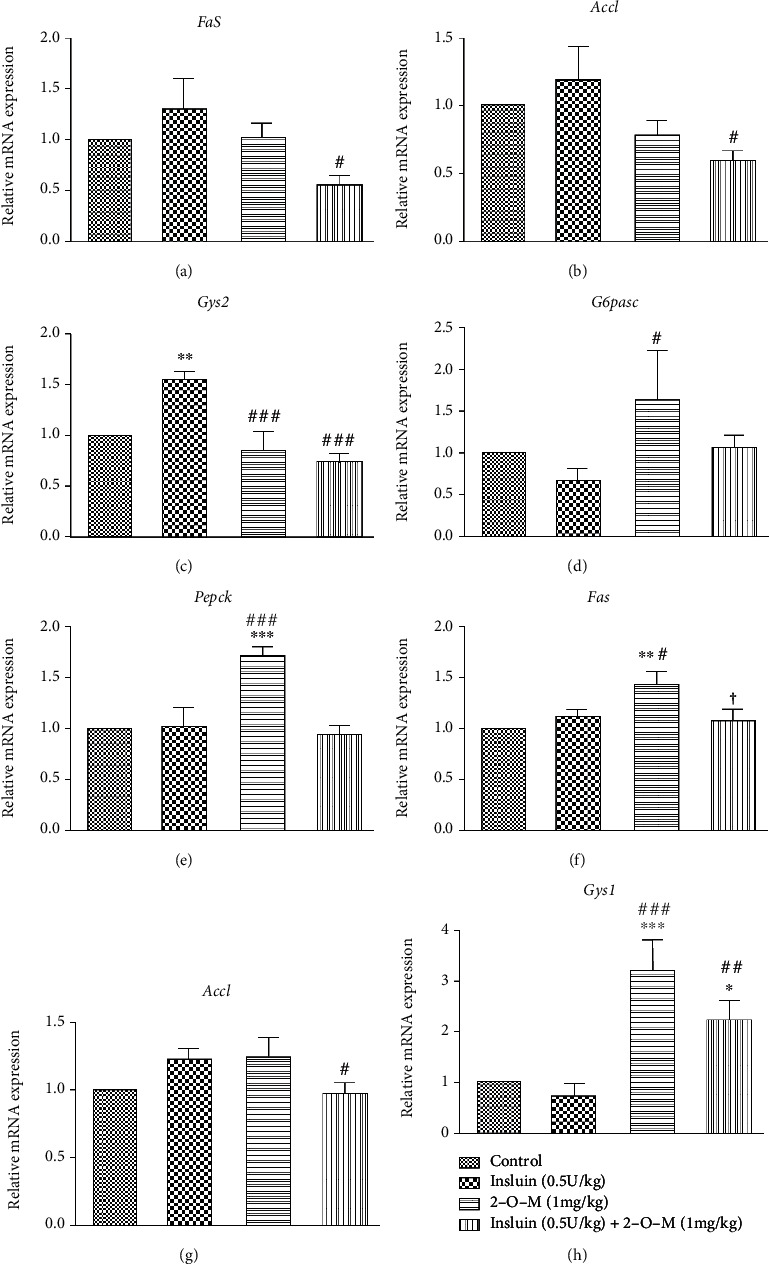
Effect of 2′-O-methylperlatolic acid (2-O-M) on the insulin signal pathway in muscle and liver tissues of diabetic mice. (a) Expression of key proteins of insulin signaling pathway in liver. (b) Expression of key proteins of insulin signaling pathway in muscle. Data are shown as the mean ± SEM (*n* = 4–8 in each group). ^∗^*p* < 0.05; ^∗∗^*p* < 0.01; ^∗∗∗^*p* < 0.001 versus control. ^#^*p* < 0.05; ^##^*p* < 0.01; ^###^*p* < 0.001 versus insulin. ^†^*p* < 0.05; ^††^*p* < 0.01, 2-O-M versus insulin+2-O-M.

The combination of 2-O-M and insulin can significantly enhance the hypoglycemic effect of insulin in diabetic mice and warrants additional exploration to reveal the specific mechanism(s) and the fate of glucose *in vivo*. Therefore, we investigated the expression of proteins related to the insulin signaling pathway in the liver and muscle tissues of the mice 120 min after treatment. The phosphorylation of the InsR and AKT in the combined treatment group was significantly lower in the liver tissues than in the insulin group (Figure 5(a)). However, compared to the insulin group, phosphorylation of the InsR and AKT in the combined treatment group was significantly increased in muscle tissues (Figure 5(b)). In addition, phosphorylation of AKT in the 2-O-M group was significantly higher than in the other three groups (Figure 5(b)). In *db/db* mice, the key proteins of the insulin signaling pathway in muscles were detected after injection of 2-O-M. We found that the combination of 2-O-M and insulin could significantly activate InsR and AKT phosphorylation. Further, phosphorylation of AKT in the 2-O-M group was also significantly higher than that of the control group and the insulin group.

### 3.4. Effect of 2-O-M on Glycogen Synthesis, Fat Synthesis, and Gluconeogenesis Genes in Diabetic Mice

**Figure 6:**
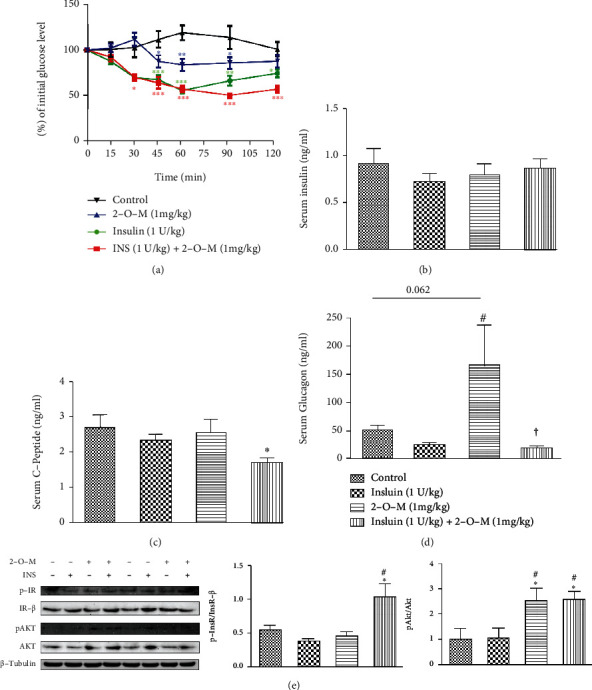
The effect of 2′-O-methylperlatolic acid (2-O-M) on glycogen synthesis, fat synthesis, and gluconeogenesis genes *in vivo*. (a–e) Genes in the liver. Genes in the muscle. Data are shown as the mean ± SEM (*n* = 4–8 in each group). ^∗^*p* < 0.05; ^∗∗^*p* < 0.01; ^∗∗∗^*p* < 0.001 versus control. ^#^*p* < 0.05; ^##^*p* < 0.01; ^###^*p* < 0.001 versus insulin. ^†^*p* < 0.05; ^††^*p* < 0.01, 2-O-M versus insulin+2-O-M.

**Figure 7:**
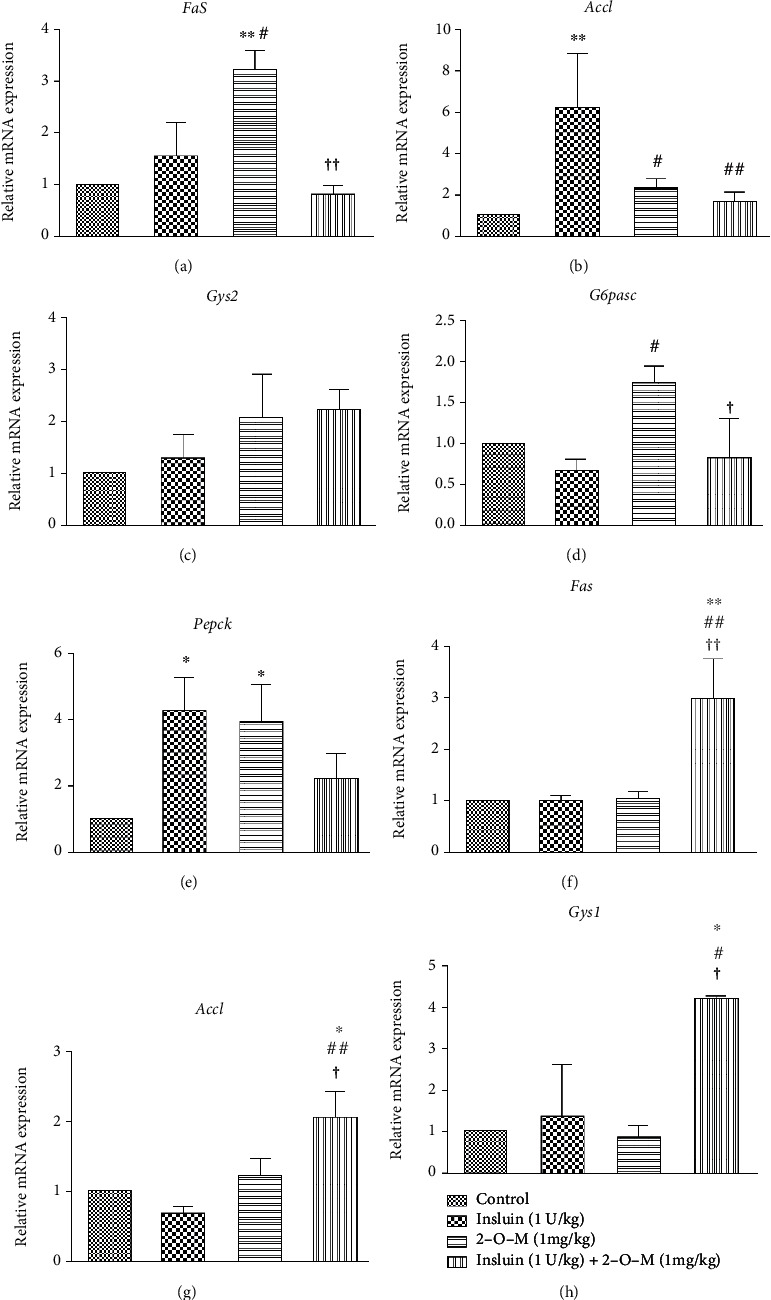
The effect of 2′-O-methylperlatolic acid (2-O-M) on glycogen synthesis, fat synthesis, and gluconeogenesis genes in *db/db* mice. (a–e) Genes in the liver. (f–h) Genes in the muscle. Data are shown as the mean ± SEM (*n* = 6 in each group). ^∗^*p* < 0.05; ^∗∗^*p* < 0.01 versus control. ^#^*p* < 0.05; ^##^*p* < 0.01 versus insulin. ^†^*p* < 0.05; ^††^*p* < 0.01, 2-O-M versus insulin+2-O-M.

To explore whether 2-O-M weakened the effect of insulin on lipid metabolism and glucose metabolism in the liver and muscle tissues of mice, we determined the mRNA expression of genes related to lipid metabolism and glucose metabolism after 2 h of treatment with 2-O-M. In the liver tissues, expressions of lipid synthesis genes *Fas* and *Acc1* were significantly decreased in the combination group with the insulin group, whereas insulin tended to enhance the expression of fat synthesis genes (Figures 6(a) and 6(b)). In the *db/db* mouse model, 2-O-M significantly enhanced *Fas* gene expression and inhibited *Acc1* gene expression (Figures 7(a) and 7(b)). The detection of glycogen synthesis gene expression revealed that the relative expression level of *Gys2* was significantly reduced in the combination group, and the expression levels of *Gys2* were similar between the control and the 2-O-M groups (Figure 6(c)). However, in *db/db* mice, there was no difference in *Gys2* expression among all groups (Figure 7(c)). In addition, the genes involved in gluconeogenesis (*G6pase* and *Pepck*) in two mouse models of diabetes were significantly enhanced in the 2-O-M group (Figures 6(d), 6(e), 7(d), and 7(e)). In the muscle tissues, the expression of fat synthesis genes *Fas* was significantly enhanced in the 2-O-M group. Compared to the insulin group, the expression of *Acc1* was significantly decreased in the combination group. Expression of *Fas* and *Acc1* in the combined group was significantly higher compared to other groups (Figures 7(f) and 7(g)). In both diabetic mice models, the expression of *Gys1* in the combination group was significantly enhanced, and the difference was that in the STZ-induced model, the expression of *Gys1* in the combined group was also significantly enhanced.

## 4. Discussion

The InsR is an important target for glycemic control [18, 19]. A previous review suggests that all InsR domains have the potential to be drug targets [20, 21]. Insulin sensitizers represent a new type of therapeutic drug for diabetes patients. Insulin sensitizers bind allosterically to InsR and sensitize insulin action to alleviate insulin resistance and minimize the hypoglycemia risk. However, insulin, insulin mimics, or orthosteric InsR activators may increase the risk of hypoglycemia in patients with diabetes, potentially leading to worse glycemic control in patients. Therefore, these factors represent limitations for the wider adoption of insulin products or InsR activators. Until now, only two InsR sensitizers, TLK19781 [19] and TLK16998 [20, 21], have been reported. Therefore, it would be beneficial to discover new small molecular natural compounds that can act as insulin sensitizers for use as compliments to insulin for patients with diabetes.

In this study, we screened 400 small molecular natural compounds using SPR and found that 2-O-M binds to the extracellular domain of the InsR with the highest response value (results not shown). Further studies found that 2-O-M has the potential to bind to the extracellular domain of the InsR protein with a *K*_*D*_ value of 88.72 *μ*M (Figure 1(b)) and that the dose range of 2-O-M used in the experiment was not toxic to cells (Figure 1(c)). In C2C12 and Hepa 1-6 cell lines, we used insulin as a positive control, and the insulin signaling pathway was significantly activated following treatment with 1 nM 2-O-M for 20 min. We also found that 2-O-M cannot activate the insulin signaling pathway but could enhance the function of insulin, significantly increasing the expression of the insulin signaling pathway and improving glucose uptake (Figure 2). These results indicate that 2-O-M directly binds to the InsR but cannot activate the insulin signaling pathway alone and that 2-O-M can significantly enhance the insulin-activated insulin signaling pathway to substantially enhance the glucose uptake capacity of cells. Furthermore, in this study, the combination of insulin with 2-O-M resulted in improved glucose-lowering effects compared to insulin alone, even though 2-O-M alone did not appear to exert a glucose-lowering effect (Figure 3(a)). Thus, 2-O-M acts as an insulin sensitizer, assisting insulin to activate the insulin signaling cascade. Previous studies have reported that several InsR sensitizers, such as TLK16998, TLK19781, and dicholine succinate, did not activate insulin signaling but could enhance the action of insulin on InsR phosphorylation [19, 21]. Because of their potential to alleviate insulin resistance and minimize the risk of hypoglycemia, insulin sensitizers represent a complementary therapeutic approach for diabetes [19].

In this study, the combined injection of insulin and 2-O-M into diabetic mice showed that 2-O-M could effectively prolong the glucose-lowering effect of insulin *in vivo* (Figure 3(a)). An examination of the serum concentrations of insulin, C-peptide, and glucagon 120 min after their injection into mice revealed that average insulin levels were similar among groups, but the average level of C-peptide was significantly reduced in the combined treatment group (Figures 3(b) and 3(c)). Exogenous insulin can significantly inhibit the secretion of endogenous insulin [24]. This response was observed in the mice treated with insulin only. Conversely, in the mice treated with both insulin and 2-O-M, the inhibition of endogenous insulin secretion was observed, in contrast to the insulin response in the mice treated with insulin alone (Figure 3). Serum levels of insulin, glucagon, and C-peptide in *db/db* mice were detected, and the conclusion was the same as that in STZ-induced diabetic mice (Figures 4(b). These results indicate that 2-O-M can improve the impact of exogenous insulin *in vivo* and further reduce endogenous insulin secretion. Therefore, combined administration of 2-O-M with insulin not only exerts a better glucose-lowering effect, minimizing the risk of hypoglycemia, but also decreases the secretion of endogenous insulin to protect *β* cell function.

As shown in Figure 3(a), the blood glucose level of the insulin group had returned to the initial level at 120 min, so no difference was found in the levels of phosphorylated AKT and InsR in liver and muscle tissues isolated from the insulin group (Figure 5). Protein expression analysis in liver tissues revealed that levels of phosphorylated AKT in the combined group and in the 2-O-M-treated group were significantly reduced (Figure 5(a)). However, in muscle tissue, the insulin signaling pathway was significantly activated in the combined group and significantly enhanced the accumulation of glycogen in the muscle tissues of diabetic mice (Figure 5(b)). In the muscle tissue of *db/db* mice, the levels of phosphorylated AKT and InsR in the 2-O-M combined with insulin group were significantly increased, showing that 2-O-M treatment directly activates muscle tissue AKT phosphorylation. In addition, 2-O-M played very different roles in the liver and muscle tissues of diabetic mice, demonstrating tissue heterogeneity that might reflect the apparent heterogeneity of InsR structure in different tissues [25].

In STZ-induced diabetic mice, the expression of *Acc1* and *Fas* was increased in the insulin-treated group. While this was not significant, the expression of these genes was significantly inhibited in the liver tissues in the combined treatment group (Figures 6(a) and 6(b)). However, the expression of *Acc1* and *Fas* increased significantly in the 2-O-M group in muscle tissues, which was consistent with the high activation of AKT in muscles (Figure 5(b)). These results showed that the combination of insulin with 2-O-M could inhibit fat synthesis in both liver and muscle tissues, which might help reduce lipid accumulation *in vivo*. Therefore, further studies should focus on the mechanisms of inhibition of insulin-induced lipogenesis by 2-O-M. A previous study has shown that increased expression of phosphorylated AKT causes membrane transfer of the glucose transporter and promotes glucose uptake [26]. In *db/db* mice, 2-O-M combined with insulin still significantly inhibited the expression of *Fas* and *Acc1* in the liver. In muscle, however, 2-O-M combined with insulin enhanced the expression of *Fas* and *Acc1*. This may be related to different mechanisms of lipid metabolism in the two animal models. In the two diabetes model mice, *Gys1* in the muscle of the mice in the combination group was significantly enhanced, indicating that the combination of 2-O-M and insulin can significantly enhance the glycogen synthesis of the muscle in both models. But the difference is that 2-O-M treatment alone can also significantly increase the expression of *Gys1* gene in muscle in STZ-induced diabetic mice, indicating that 2-O-M exhibits different mechanisms of action in the two model mice 7(h)).

In this study, we found that 2-O-M activates phosphorylation of AKT in muscle tissues, but not through activation of the InsR (Figure 5(b)). In addition, 2-O-M showed no effect on lowering blood glucose in diabetic mice (Figure 3(a)). These results imply that other mechanisms may exist to drive the 2-O-M activation of AKT phosphorylation. We also found that the concentration of serum glucagon in mice in the 2-O-M group was significantly higher than the concentration in other groups (Figures 3(c) and 6(d)). We determined the mRNA expression of genes involved in gluconeogenesis (*G6pase* and *Pepck*) in the liver tissues of STZ-induced diabetic mice and found that the expression of these genes significantly increased in the 2-O-M group (Figures 6(d) and 6(e)). We also found the same results in *db/db* mice (Figures 7(d) and 7(e)). These data suggest that 2-O-M increases glucagon secretion and induces gluconeogenesis in the liver tissues of diabetic mice. Glucagon and insulin are completely competitive antagonists *in vivo* [27]. Therefore, 2-O-M may activate both AKT phosphorylation and gluconeogenesis *in vivo*; this hypothesis is supported by the lack of a glucose-lowering effect by 2-O-M *in vivo*.

## 5. Conclusions

SPR experiments demonstrate that 2-O-M binds to the extracellular domain of InsR. The combination of 2-O-M and insulin significantly activates the insulin signaling pathway *in vitro* and significantly stimulates the glucose uptake capacity of C2C12 myotubes. Further, 2-O-M significantly reduces the secretion of endogenous insulin and reduces the blood glucose concentration *in vivo*. In addition, 2-O-M alone significantly enhances the phosphorylation level of AKT in muscle tissue, which also enhances glucose uptake in C2C12 myotubes. In contrast, 2-O-M significantly enhances the secretion of glucagon and enhances liver gluconeogenesis to mitigate the risk of hypoglycemia. Therefore, 2-O-M can be used as a supplement to insulin, allowing the injection of lower doses of insulin, which may reduce the potential adverse effects of injected insulin (e.g., hypoglycemia and obesity) to protect *β* cell function.

## Figures and Tables

**Table 1:** Gene sequences of primers.

Gene name	Forward primer	Reverse primer
*β*-Actin	5′-GAGACCTTCAACACCCCAGC-3′	5′-ATGTCACGCACGATTTCCC-3′
Gys	5′-ATCTTCTTCGTCTTCCGCATC-3′	5′-GACACTGAGCAGGGCTTTTCC-3′
G6pase	5′-AAAAAGCCAACGTATGGATTCCG-3′	5′-CAGCAAGGTAGATCCGGGA-3′
Pepck	5′-TTTGATGCCCAAGGCAACTT-3′	5′-ATCGATGCCTTCCCAGTAAA-3′
Fas	5′-CTGGCATTCGTGATGGAGTC-3′	5′-TGTTTCCCCTGAGCCATGTA-3′
Acc1	5′-CGCTCGTCAGGTTCTTATTG-3′	5′-TTTCTGCAGGTTCTCAATGC-3′

*α*-Tubulin	5′-CTCTACCCTCACATCCACTTCC-3′	5′-ATAGGCAGCAAGCCATGTATTT-3′

## Data Availability

The data analyzed during the study are not publicly available. Rigorous analysis of the data in order to ensure the objective authenticity of the results.

## Supplementary Materials

Supplementary Materials Figure S1: change in body weight (A) and blood glucose concentration (B) of mice after STZ injection. ^∗^*p* < 0.05; ^∗∗^*p* < 0.01; ^∗∗∗^*p* < 0.001 versus day 0.
