# Supplementary material for: 2′-O-Methylperlatolic Acid Enhances Insulin-Regulated Blood Glucose-Lowering Effect through Insulin Receptor Signaling Pathway
Source: J Diabetes Res. 2022 Apr 23;2022:2042273. doi: 10.1155/2022/2042273 (PMC9056246; doi:10.1155/2022/2042273)
Supplement: Supplementary Materials — Figure S1: change in body weight (A) and blood glucose concentration (B) of mice after STZ injection. ∗p < 0.05; ∗∗p < 0.01; ∗∗∗p < 0.001 versus day 0. [file 2042273.f1.docx]

**A**

**B**

**Fig. S1. Change in body weight (A)** and blood glucose concentration **(B)** of mice after STZ injection. * *p*<0.05; ** *p*<0.01; *** *p*<0.001 versus day 0.
